# Efficacy and Safety of Celecoxib and a Korean SYSADOA (JOINS) for the Treatment of Knee Osteoarthritis: A Systematic Review and Meta-Analysis

**DOI:** 10.3390/jcm14041036

**Published:** 2025-02-07

**Authors:** Yong-Beom Park, Jun-Ho Kim

**Affiliations:** 1 Department of Orthopedic Surgery, Chung-Ang University Gwangmyeong Hospital, Chung-Ang University College of Medicine, Seoul 14353, Republic of Korea; whybe1122@gmail.com; 2 Department of Orthopedic Surgery, Hallym Sacred Heart University Hospital, Hallym University, Anyang-si 13496, Republic of Korea

**Keywords:** celecoxib, COX-2 inhibitor, SKCPT, SKI306X, knee osteoarthritis

## Abstract

**Background:** The efficacy of cyclooxygenase-2 (COX-2) inhibitors, including celecoxib, in managing knee osteoarthritis (KO) is well-established. Recently, the plant extract cocktail JOINS (SKI306X and its newer formulation, SKCPT) has been shown to be an effective slow-acting drug for KO. **Aims:** To compare the efficacy and safety of celecoxib and JOINS in patients with KO. **Methods:** A systematic search of the MEDLINE, Embase, and Cochrane Library databases identified randomized controlled trials (RCTs) assessing the effectiveness and safety of celecoxib and JOINS. The outcomes included pain relief, functional improvement, and safety profiles. Outcome measurements were compared between the celecoxib and JOINS cohorts at the short-term (closest to 3 months) and mid-term (closest to 12 months). **Results:** Overall, 23 RCTs involving 3367 patients were included in this systematic review. The efficacy of JOINS in reducing pain, as indicated by the visual analog scale (VAS) score, was comparable to that of celecoxib. Regarding functional improvement assessed using the Western Ontario and McMaster University Arthritis Index (WOMAC), JOINS showed improvement comparable to that of celecoxib regardless of follow-up. In addition, no significant difference was observed in the incidence of adverse events between the celecoxib and JOINS cohorts. **Conclusions:** The results of this study suggest that JOINS could be considered as a pharmacological agent with significant efficacy for pain relief and functional improvement in patients with KO in clinical practice.

## 1. Introduction

Knee osteoarthritis (KO) is a common chronic degenerative disease that causes joint pain, tenderness, limited movement, and reduced quality of life. With an aging population and a global increase in obesity, the incidence of KO is increasing annually, making it a significant challenge for health systems worldwide [1,2,3,4]. OA is the seventh leading cause of disability in adults aged ≥ 70 years [1]. KO is quite common in Korea, with a prevalence of 33.3–37.3% among those aged ≥ 50 years [5,6]. Given the lack of curative treatments for OA, current strategies focus on alleviating pain and minimizing functional limitations. Although nonsteroidal anti-inflammatory drugs (NSAIDs) are commonly used to manage KO, their long-term use is associated with an increased risk of gastrointestinal and cardiovascular events [7,8]. Therefore, symptomatic slow-acting drugs for OA (SYSADOAs), including glucosamine, chondroitin, diacerein, avocado soybean unsaponifiables, and herbal medicines, are commonly used worldwide [9,10,11].

Herbal medications are frequently prescribed in Asian countries in addition to in the US and in European countries [12,13]. SKI306X/SKCPT (JOINS^®^) is currently the most prescribed SYSADOA in Korea. It is formulated from the ethanol extract of three medicinal plants: *Clematis mandshurica*, *Trichosanthes kirilowii*, and *Prunella vulgaris* [12]. Several preclinical and clinical studies have demonstrated the efficacy and safety of herbal medicine [12,13,14,15,16,17,18,19].

Therefore, in this study, we aimed to provide clinical evidence regarding the efficacy and safety of JOINS in patients with KO to help inform clinical practice regarding using SYSADOAs for KO. We hypothesized that JOINS would be comparable to celecoxib in providing pain relief and improving KO function.

## 2. Materials and Methods

The systematic review was developed following Cochrane review methods and adhered to the PRISMA guidelines for systematic reviews and meta-analyses [20]. The study protocol was registered in the International Prospective Register of Systematic Reviews (registration no.: CRD42024573087).

### 2.1. Literature Search

A comprehensive literature search was conducted utilizing the PubMed (MEDLINE), Embase, and Cochrane Library databases up to 1 September 2024, with no restrictions on language or year of publication. A combination of the following keywords was used in the title, abstract, Medical Subject Heading, and keywords fields: (“celecoxib” OR “Celebrex” OR “COX-2 inhibitor” “SKCPT” OR “SKI306X”) AND (“knee”) AND (“osteoarthritis”). The research questions and inclusion criteria were defined beforehand. Manual searches were conducted for articles that may have been overlooked during the electronic search. Celecoxib was selected because it is widely used as a control drug in most studies [21,22]. Including other NSAIDs could introduce additional heterogeneity, thereby confounding the results. For this reason, only celecoxib was included in the analysis. The bibliographies of the initially retrieved studies were meticulously cross-checked to identify additional relevant articles. Two reviewers, Y-BP and J-HK, independently conducted a thorough screening of the abstracts and titles of these studies. The studies that met the established inclusion criteria subsequently underwent a comprehensive full-text review.

### 2.2. Study Selection

Two reviewers (Y-BP and J-HK) independently screened the titles and abstracts of the retrieved articles. When the abstract did not contain relevant data for inclusion in the study, the entire manuscript was reviewed. Disagreements were resolved through discussion. Studies were included in the current systematic review based on meeting the patient, intervention, comparison, outcome, and study design criteria (Table 1) [23]. Randomized controlled trials (RCTs) of patients with KO of Kellgren–Lawrence (K–L) grades I, II, or III who were treated with celecoxib or JOINS (SKCPT or SKI306X) vs. placebo or non-placebo, such as NSAIDs or other SYSADOAs, were included in the current systematic review. The exclusion criteria were as follows: (1) conference or (2) clinical trial abstracts; (3) insufficient statistics or inability to reproduce statistics; (4) in vitro studies; and (5) levels of evidence grade III, IV, or V.

### 2.3. Assessments of Methodological Quality

Two investigators, Y-BP and J-HK, assessed each study’s quality using the methodological index for non-randomized studies (MINORS) criteria [24], with maximum scores of 24 for comparative studies, according to the MINORS checklist [24]. Moreover, the two reviewers assessed the risk of bias in the included RCTs using the Cochrane Handbook for Systematic Reviews of Interventions [25]. This tool evaluates bias across the following several domains: selection, performance, detection, and attrition. Disputes and differences in scores between the two reviewers were addressed through discussion.

### 2.4. Data Extraction and Synthesis

Two dedicated investigators, Y-BP and J-HK, meticulously extracted data from each article using a carefully designed data extraction form, ensuring accuracy and consistency throughout the process. Disagreements were approached as opportunities for constructive dialogue, leading to effective resolutions. The collected data included the study characteristics (author, year of publication, country, and sample size), patient characteristics (mean age, sex proportion, indication, and OA grading), and management details (intervention type, daily dose, treatment duration, follow-up, and rescue medicine). Outcome measures for pain (100 mm VAS score), function (Western Ontario McMaster University Arthritis Index [WOMAC]), and safety (adverse events [AEs], adverse drug reactions [ADRs], and serious adverse events [SAEs]) were recorded using a predefined data form; ADR was considered to have a causal relationship with the drugs. In case of missing data, we first attempted to contact the authors. After the initial approach failed, we estimated the missing values using the methods outlined in the Cochrane Handbook for Systematic Reviews of Interventions [25].

### 2.5. Statistical Analysis

The main objective of this meta-analysis was the indirect comparison of celecoxib with JOINS in terms of pain relief, functional improvement, and safety. Clinical outcomes were assessed by comparing post-medication values with pre-medication values using formulas from the Cochrane Handbook for Systematic Reviews of Interventions [25] and were analyzed for short-term (closest to 3 months) and mid-term (closest to 12 months) follow-ups. When feasible, a single-arm meta-analysis was conducted to calculate the effect size of the mean difference with 95% confidence intervals (CIs) for continuous variables and the odds ratio (OR) with 95% CIs for dichotomous variables. A qualitative description of the outcomes was provided when a meta-analysis could not be performed owing to insufficient data. A random-effects model utilizing the restricted maximum likelihood method was employed to synthesize outcomes across the included studies. This method yields more dependable conclusions for diverse patient populations and various surgical procedures [26,27]. We used forest plots to display the results of each study and to show the combined effect. These plots were created using Open Meta-Analyst from Brown University (http://www.cebm.brown.edu/openmeta, accessed on 1 September 2024). The standardized mean difference (d) and variance (vd) were calculated using the logit method based on each cohort’s weighted estimates, standard errors, and sample sizes [28,29]. Summary ORs and 95% CIs were calculated based on d and vd (George Wilson University, Fairfax, VA, USA). Publication bias was not assessed, as it is not required with fewer than 10 studies [25]. Statistical significance was defined as a *p* value of less than 0.05.

## 3. Results

### 3.1. Study Identification

**Figure 1:**
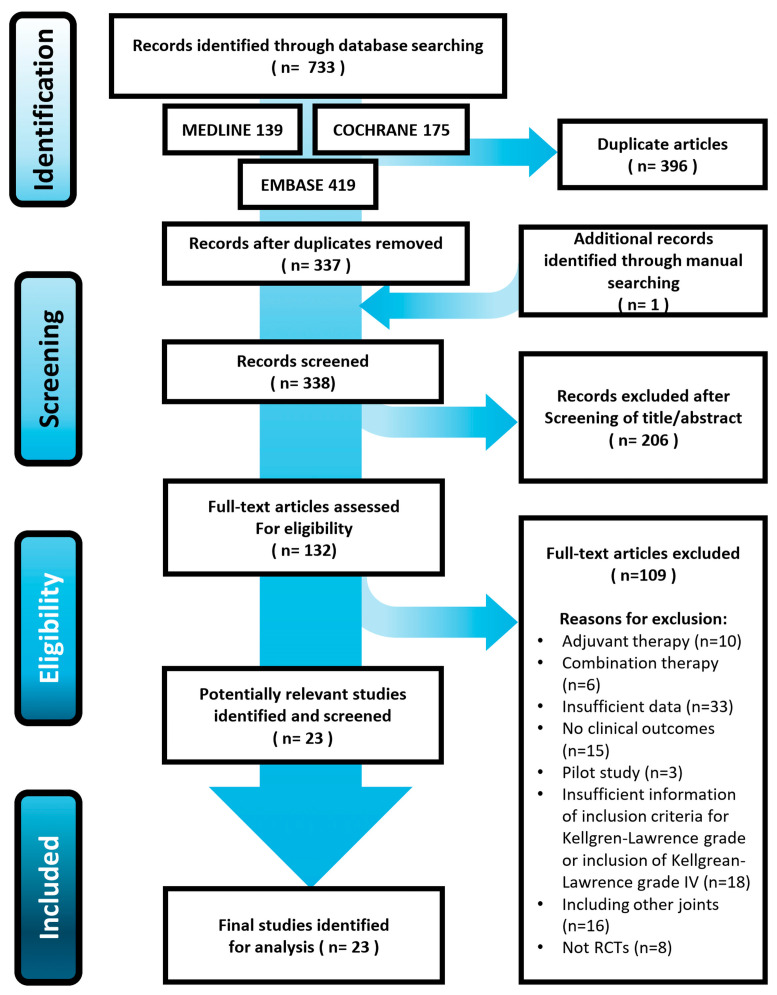
Preferred Reporting Items for Systematic Reviews and Meta-analyses (PRISMA) flow diagram for identifying and selecting the studies included in this meta-analysis.

The initial electronic search resulted in 733 studies; after removing 396 duplicates, 337 studies remained. After screening the titles and abstracts and reading the full text, 23 RCTs were included in this systematic review. Figure 1 presents the details of the study identification, inclusion, and exclusion criteria.

### 3.2. Study Characteristics and Methodological Quality Assessment

One [18] of the twenty-three identified studies directly compared celecoxib and JOINS treatments. Nineteen studies [18,22,30,31,32,33,34,35,36,37,38,39,40,41,42,43,44,45,46] involving 2989 knees evaluated celecoxib treatment, whereas five [12,15,17,18,19] studies involving 378 knees assessed JOINS treatment (Table 2). Of the 23 RCTs, 19 [12,15,18,19,22,30,31,33,34,35,36,37,38,39,41,42,43,44,46] reported clinical outcomes at short-term follow-up, and 11 [12,17,18,30,32,37,39,40,41,42,45] reported mid-term outcomes (Table 3). Methodological quality assessment using MINORS showed pooled median scores of 23 (range, 21–24) and 23 (range, 22–24) in the celecoxib and JOINS groups, respectively (Supplementary Table S1). The risk of bias was generally low, with only two studies having a high risk of bias for the items ‘blinding of outcome assessment’ [15] and ‘other bias’ [35] (Supplementary Table S2).

### 3.3. Clinical Effectiveness

#### 3.3.1. Pain Relief

**Figure 2:**
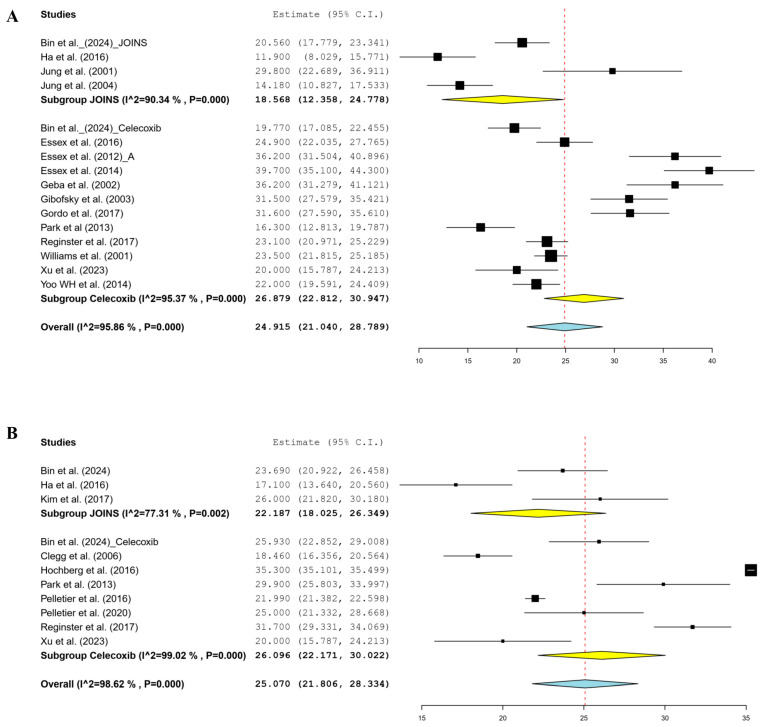
Forest plots of included studies showing changes in the 100 mm VAS for pain relief in the short- (**A**) and mid-term (**B**) follow-up periods before and after knee osteoarthritis treatment using celecoxib and JOINS. Squares represent the mean change in outcomes, with the size of the square being proportional to the sample size. VAS, visual analog scale; CI, confidence interval. Squares and redline represent the mean change in outcomes, with the size of the square being proportional to the sample size [12,17,18,22,30,31,32,34,35,36,37,39,40,41,42,43,44,46].

In the short-term follow-up, twelve celecoxib [18,22,31,33,34,35,36,39,42,43,44,46] and four JOINS [12,15,18,19] cohorts reported changes in the 100 mm VAS scores from the preoperative to postoperative periods. The meta-analysis-estimated pain relief in the 100 mm VAS was 26.9 (95% CI, 22.8–30.9) in the celecoxib and 18.6 (95% CI, 12.4–24.8) in the JOINS cohorts, which was not significantly different (*p* = 0.108) (Figure 2A). In the mid-term follow-up, seven celecoxib [18,30,37,39,40,42,44] and three JOINS [12,17,18] cohorts showed a mean improvement in the 100 mm VAS of 26.1 (95% CI, 22.2–30.0) and 22.2 (95% CI, 18.1–26.4), respectively, which indicated no significant difference between the two cohorts (*p* = 0.458) (Figure 2B).

#### 3.3.2. Functional Improvement

**Figure 3:**
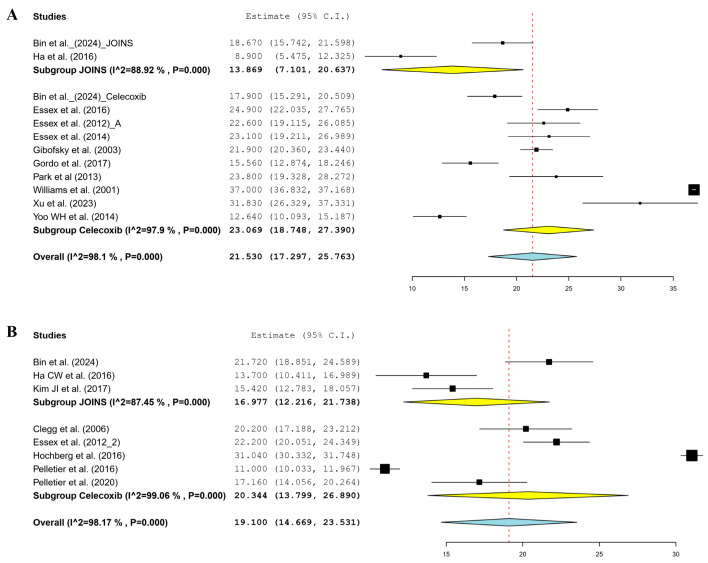
Forest plots of included studies showing changes in the total WOMAC score for functional improvement in the short- (**A**) and mid-term (**B**) follow-up periods before and after knee osteoarthritis treatment using celecoxib and JOINS. Squares represent the mean change in outcomes, with the size of the square being proportional to the sample size. VAS, visual analog scale; CI, confidence interval; WOMAC, Western Ontario McMaster University Arthritis Index. Squares and redline represent the mean change in outcomes, with the size of the square being proportional to the sample size [12,17,18,30,31,32,33,34,35,36,37,39,40,41,43,44,46].

In the short-term follow-up, ten celecoxib [18,31,33,34,35,36,39,43,44,46] and two JOINS [12,18] cohorts reported changes in the total WOMAC score from the preoperative to postoperative period. The meta-analysis-estimated functional improvement of the total WOMAC score was 23.1 (95% CI, 18.8–27.4) in the celecoxib and 13.9 (95% CI, 7.1–20.6) in the JOINS cohorts, which indicated no significant difference between the two cohorts (*p* = 0.159) (Figure 3A). In the mid-term follow-up, seven celecoxib [18,30,32,37,40,41,44] and three JOINS [12,17,18] cohorts reported a mean improvement in total WOMAC score of 20.3 (95% CI, 13.8–26.9) and 17.0 (95% CI, 12.2–21.7), respectively, which indicated no significant difference between the two cohorts (*p* = 0.451) (Figure 3B).

### 3.4. Safety

**Figure 4:**
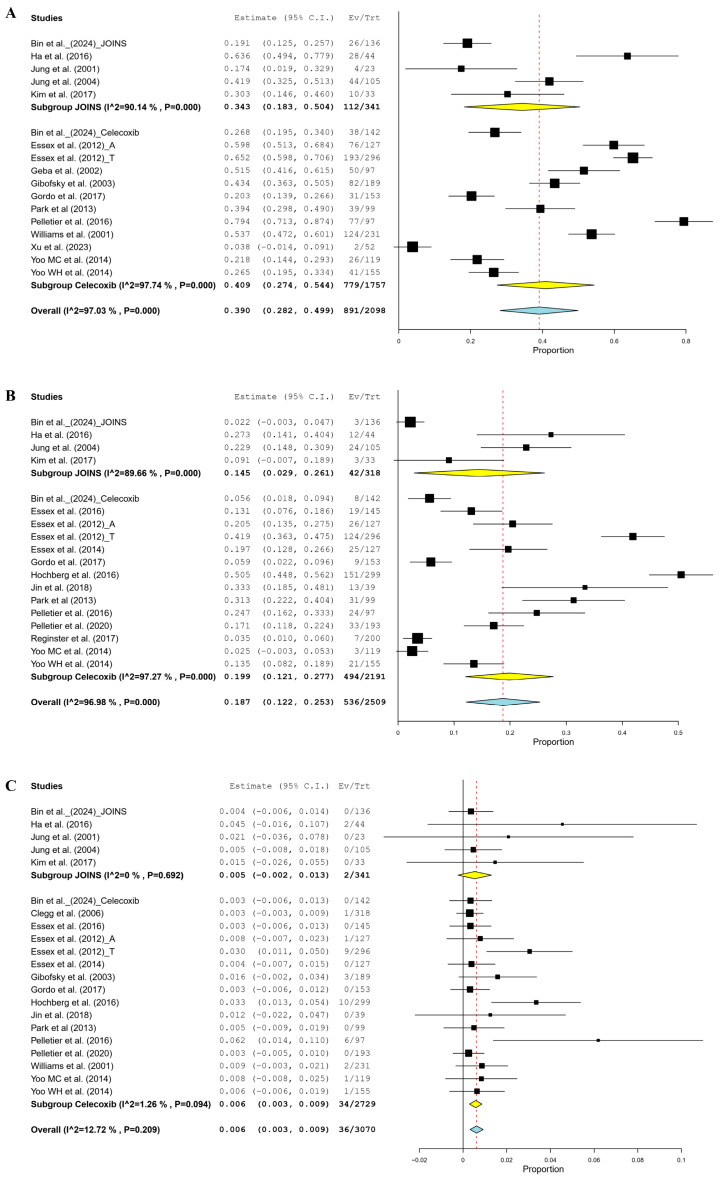
Forest plots of studies showing the pooled rate of all adverse events (**A**), adverse drug reactions (**B**), and serious adverse events (**C**) between celecoxib and JOINS in patients with knee osteoarthritis. The squares represent the rate of adverse events after treatment with celecoxib and JOINS in patients with knee osteoarthritis, with the size of the square proportional to the sample size. CI, confidence interval; Ev/Trt, events/treatment. Squares and redline represent the mean change in outcomes, with the size of the square being proportional to the sample size [12,15,17,18,19,22,31,32,33,34,35,36,37,38,39,40,41,42,43,44,45,46].

The meta-analysis estimated that the rate of all AEs was 39.0% (95% CI, 28.2–49.9%); 40.9% in the celecoxib group and 34.3% in the JOINS group, showing no significant difference between the two cohorts (*p* = 0.681) (Figure 4A). Furthermore, the rate of ADRs was estimated to be 18.7% (95% CI, 12.2–25.3%), 19.9% in the celecoxib and 14.5% in the JOINS cohorts, showing no significant difference between the two (*p* = 0.615) (Figure 4B). The SAE rates were not significantly different between the celecoxib and JOINS cohorts (0.6% and 0.5%, respectively) (Figure 4C).

## 4. Discussion

To the best of our knowledge, this is the first meta-analysis comparing Korean SYSADOA JOINS vs. celecoxib to evaluate their efficacy and safety in patients with KO. In this study, the efficacy of JOINS, a Korean SYSADOA, in treating KO was systematically evaluated by patients in terms of VAS and WOMAC scores. Based on the results of this meta-analysis, JOINS demonstrated good efficacy for pain relief and functional improvement in patients with KO, comparable to that of celecoxib. To clarify the effects of JOINS, studies involving only celecoxib were included as a control group.

Although some guidelines do not recommend SYSADOAs for KO treatment, the European Society for Clinical and Economic Aspects of Osteoporosis and Osteoarthritis (ESCEO) guidelines endorsed SYSADOAs (glucosamine sulfate and chondroitin sulfate) as first-line treatments for KO. The results of this meta-analysis were consistent with the ESCEO guidelines. Therefore, the Korean SYSADOA JOINS is a reasonable pharmacological option for managing KO in clinical practice.

The current meta-analysis showed that JOINS was clinically effective in pain relief and functional improvement in KO. At the short-term follow-up (≤3 months), JOINS showed a significant reduction in pain, as measured using the 100 mm VAS score, which was comparable to that of celecoxib. In addition, at the mid-term follow-up (>3 months), JOINS showed a significant improvement in pain, comparable to that of celecoxib. There were no significant differences in functional improvement between the celecoxib and JOINS cohorts, regardless of the follow-up period. Celecoxib, a selective COX-2 inhibitor, provides anti-inflammatory and analgesic effects and is an effective therapy widely used for treating KO [47]. Collectively, the findings of this meta-analysis and previous studies suggest that the Korean SYSADOA JOINS is an effective treatment option for pain management and functional improvement in patients with KO.

No significant differences in AEs were observed between the celecoxib and JOINS cohorts. This may be attributed to the type and duration of NSAIDs used. NSAIDs are significantly associated with gastrointestinal and cardiovascular complications [7,48]. Celecoxib decreased gastrointestinal complications but did not increase cardiovascular complications compared to traditional NSAIDs. [49,50] In addition, among the included studies, 12 (63.2%) had patients with short-term use of celecoxib (<3 months). A recent study reported a 36–50% reduction in the need for concomitant NSAIDs in patients prescribed with SYSADOAs [51]. Additionally, another study reported that SYSADOAs, including JOINS, contributed to the discontinuation of NSAIDs in patients with KO [52]. Collectively, these findings suggest that JOINS is an effective and safe treatment option for KO.

According to a Korean nationwide claims database, SYSADOAs are widely used in Korea, with 43.4% of the patients using one or more drugs to treat OA [53]. SKCPT/SKI306X (JOINS^®^) is a Korean herbal SYSADOA product formulated from a 30% ethanol dry extract of *Clematis mandshurica*, *Trichosanthes kirilowii*, and *Prunella vulgaris*, plants that have been widely used for treating inflammatory diseases in East Asia [18]. Preclinical studies have reported positive biological effects, including anti-inflammatory actions through the suppression of proinflammatory cytokine expression and cartilage-protective effects via the regulation of tissue inhibitors of metalloproteinases and matrix metalloproteinase production [13,54,55]. Clinical trials have demonstrated a similar pain relief and functional improvement as that of NSAIDs, but with fewer AEs [17,18,56]. This review demonstrates that JOINS can improve pain and function in patients with KO. Therefore, this study provides robust clinical evidence supporting the use of the Korean SYSADOA JOINS for the treatment of KO.

This study had some limitations. First, the number of studies and sample sizes were relatively small, as this study focused on Korean SYSADOAs. However, to the best of our knowledge, this is the first meta-analysis on the use of the Korean SYSADOA JOINS for treating KO. Second, no long-term follow-up studies were conducted, and only two studies included more than a 12-month follow-up period, which limited the ability to assess its long-term efficacy and safety. Third, the heterogeneity observed across the included studies was a limitation of the current study. However, heterogeneity is an inevitable characteristic of the nature of the meta-analysis, and we performed a random-effect model for meta-analysis to reflect the heterogeneity. Fourth, the comparison of SYSADOAs and JOINS exclusively with celecoxib limits the generalizability and robustness of the clinical evidence supporting their use. Although the study provides valuable insights, further research involving comparisons with additional therapeutic agents is essential to reinforce and broaden the conclusions.

## 5. Conclusions

This study confirmed that the Korean SYSADOA JOINS improved pain and function in KO and is non-inferior to celecoxib for treating KO over a 12-month period. These findings support the use of JOINS as a viable option for KO treatment in clinical practice.

## Figures and Tables

**Table 1:** Inclusion and exclusion criteria based on PICO ^α^.

PICO	Inclusion Criteria	Exclusion Criteria
Population	Patients with primary knee OA of K–L grade I, II, or III	Secondary OA Primary OA with K–L grade IV Patients with other joint OA, such as hip OA
Intervention	Treatment with celecoxib, SKI036X, or SKCPT	Adjuvant therapy
Comparison	Control group including placebo, NSAIDs, or SYSADOA treatment	Adjuvant therapy
Outcomes	Primary outcome: pain for 100 mm VAS Secondary outcomes function for WOMAC Safety profile including adverse events	
Study design (LOE)	I or II	III, IV or V

^α^ PICO, population intervention comparison outcome; LOE, level of evidence; OA, osteoarthritis; K–L, Kellgren–Lawrence; NSAIDs, nonsteroidal anti-inflammatory drug; SYSADOAs, symptomatic slow-acting drugs for osteoarthritis; VAS, visual analog scale; WOMAC, Western Ontario McMaster University Arthritis Index.

**Table 2:** General characteristics of the included studies ^α^.

Study (Year)	Country	Control	Sample Size ^ß^, n	Mean Age, y	Sex, M:F	Indication (Age, Race)	K–L Grade	MINORS Score
Celecoxib Group			2989	61.1	810:2179			23
Bin et al. [18] (2024)	S. Korea	JOINS	142	61.7	29:113	>50 yrs., Asian	I, II, III	24
Clegg et al. [30] (2006)	USA	Placebo SYSADOA	318	59.4	106:212	>40 yrs., Mixed	II, III	23
Essex et al. [34] (2016)	USA	Naproxen Placebo	145	65.9	58:97	≥45 yrs., Asian	I, II, III	24
Essex et al. [31] (2014)	USA	Naproxen Placebo	127	59.6	35:92	≥45 yrs., Hispanic	I, II, III	24
Essex et al. [32] (2012)	USA	Naproxen Placebo	296	60.0	104:192	≥45 yrs., Mixed	I, II, III	22
Essex et al. [33] (2012)	USA	Naproxen Placebo	127	58.0	25:102	≥45 yrs., African American	I, II, III	23
Geba et al. [22] (2002)	USA	Refecoxib AAP	97	62.6	34:63	≥45 yrs., Mixed	I, II, III	24
Gibofsky et al. [35] (2003)	USA	Refecoxib Placebo	189	62.2	59:130	≥40 yrs., Mixed	I, II, III	24
Gordo et al. [36] (2017)	Portugal	Ibuprofen Placebo	153	62.2	42:111	≥40 yrs., Caucasian dominant	I, II, III	24
Hochberg et al. [37] (2016)	USA	SYSADOA	258	63.2	49:209	≥40 yrs., Mixed	II, III	23
Jin et al. [38] (2018)	USA	Galcanezumab Placebo	36	60.8	14:22	40–70 yrs., Mixed	II, III	23
Park et al. [39] (2013)	S. Korea	SYSADOA	99	61.9	18:81	35–80 yrs., Asian	I, II, III	23
Pelletier et al. [41] (2020)	Canada	SYSADOA	148	64.1	37:111	≥50 yrs., Mixed	II, III	23
Pelletier et al. [40] (2016)	Canada	SYSADOA	97	61.3	36:61	≥40 yrs., Mixed	II, III	23
Reginster et al. [42] (2017)	Europe	SYSADOA Placebo	200	65.5	39:160	>50 yrs., Mixed	I, II, III	22
Williams et al. [43] (2001)	USA	Placebo	231	61.3	72:159	NR, Mixed	I, II, III	21
Xu et al. [44] (2023)	China	Tuina	52	64.6	14:38	50-80 yrs., Asian	II, III	23
Yoo MC et al. [45] (2014)	S. Korea	Etoricoxib	119	62.7	15:104	≥40 yrs., Asian	I, II, III	23
Yoo WH et al. [46] (2014)	S. Korea	SYSADOA	155	62.6	24:131	≥40 yrs., Asian	I, II, III	23
JOINS Group			378	61.3	53:325			23
Bin et al. [18] (2024)	S. Korea	Celecoxib	136	61.1	34:102	>50 yrs., Asian	I, II, III	23
Ha et al. [12] (2016)	S. Korea	SYSADOA	61	65.4	7:54	40–80 yrs., Asian	II, III	24
Jung et al. [15] (2001)	S. Korea	Placebo	23	59.1	1:22	35–75 yrs., Asian	II, III	23
Jung et al. [19] (2004)	S. Korea	Diclofenac	125	60.1	9:116	35–75 yrs., Asian	II, III	22
Kim et al. [17] (2017)	S. Korea	Placebo	33	60.2	2:31	45–79 yrs., Asian	II, III	23

^α^ M, male; F, female; K–L, Kellgren–Lawrence; GS, glucosamine sulfate; CS, chondroitin sulfate; JOINS, SK, SKI036X, or SKCPT; NR, not reported; NSAID, nonsteroidal anti-inflammatory drug; SYSADOA, symptomatic slow-acting drug for osteoarthritis; MINORS, methodological index for non-randomized studies. ^ß^ Sample size was reported based on the intention-to-treat set.

**Table 3:** Detailed protocol of management for knee osteoarthritis in the included studies ^α^.

Study (Year)	Daily Dose	Treatment Duration	Follow-Up, Months	Rescue Medicine
Celecoxib Group				
Bin et al. [18] (2024)	200 mg (1 × 200 mg)	3 months	1, 2, 3	AAP (Max. 2 g/day)
Clegg et al. [30] (2006)	200 mg (1 × 200 mg)	6 months	1, 2, 4, 6	AAP (Max. 4 g/day)
Essex et al. [34] (2016)	200 mg (1 × 200 mg)	1.5 months	1.5	AAP (Max. 2 g/day)
Essex et al. [31] (2014)	200 mg (1 × 200 mg)	1.5 months	0.5, 1.5 months	NR
Essex et al. [32] (2012)	200 mg (1 × 200 mg)	6 months	6	NR
Essex et al. [33] (2012)	200 mg (1 × 200 mg)	1.5 months	1.5	AAP (Max. 2 g/day)
Geba et al. [22] (2002)	200 mg (1 × 200 mg)	1.5 months	0.5, 1, 1.5	AAP (Max. 2.6 g/day)
Gibofsky et al. [35] (2003)	200 mg (1 × 200 mg)	1.5 month	0.75, 1.5	AAP
Gordo et al. [36] (2017)	200 mg (1 × 200 mg)	1.5 month	1.5	AAP (Max. 2 g/day)
Hochberg et al. [37] (2016)	200 mg (1 × 200 mg)	6 months	1, 2, 4, 6	AAP (Max. 3 g/day)
Jin et al. [38] (2018)	200 mg (1 × 200 mg)	4 months	2	AAP (Max. 3 g/day)
Park et al. [39] (2013)	200 mg (1 × 200 mg)	3 months	1, 2, 3	AAP
Pelletier et al. [41] (2020)	200 mg (1 × 200 mg)	6 months	2, 4, 6	AAP (Max. 2 g/day)
Pelletier et al. [40] (2016)	200 mg (1 × 200 mg)	24 months	3, 6, 12, 18, 24	AAP (Max. 3 g/day)
Reginster et al. [42] (2017)	200 mg (1 × 200 mg)	6 months	1, 3, 6	AAP (Max. 3 g/day)
Williams et al. [43] (2001)	200 mg (1 × 200 mg)	1.5 months	0.5, 1.5	AAP (Max. 2 g/day)
Xu et al. [44] (2023)	200 mg (1 × 200 mg)	0.5 months	0.5, 1.5, 1	NR
Yoo MC et al. [45] (2014)	200 mg (1 × 200 mg)	3 months	3	AAP (Max. 4 g/day)
Yoo WH et al. [46] (2014)	200 mg (1 × 200 mg)	2 months	1, 2	NR
JOINS Group				
Bin et al. [18] (2024)	600 mg (2 × 300 mg)	3 months	1, 2, 3	AAP (Max. 2 g/day)
Ha et al. [12] (2016)	600 mg (3 × 200 mg)	3 months	1, 2, 3	AAP (Max. 3.9 g/day)
Jung et al. [15] (2001)	600 mg (3 × 200 mg)	1 month	0.5, 1	Not allowed
Jung et al. [19] (2004)	600 mg (3 × 200 mg)	1 month	1	Not allowed
Kim et al. [17] (2017)	600 mg (3 × 200 mg)	12 months	3, 6, 12	AAP (Max. 4 g/day)

^α^ JOINS, SK, SKI036X, or SKCPT; NR, not reported; AAP, acetaminophen; Max., maximum.

## Data Availability

The data analyzed in this study were obtained from previously published studies and are, therefore, not available as a single dataset. Individual study data can be accessed through the cited publications.

## Supplementary Materials

The following supporting information can be downloaded at: https://www.mdpi.com/article/10.3390/jcm14041036/s1, Table S1: A summary of the MINORS score in the included studies in the meta-analysis; Table S2: A summary of the bias of RCTs included in the meta-analysis.
