# Correction: Park, Y.-B.; Kim, J.-H. Efficacy and Safety of Celecoxib and a Korean SYSADOA (JOINS) for the Treatment of Knee Osteoarthritis: A Systematic Review and Meta-Analysis. *J. Clin. Med.* 2025, *14*, 1036

**DOI:** 10.3390/jcm14176223

**Published:** 2025-09-03

**Authors:** Yong-Beom Park, Jun-Ho Kim

**Affiliations:** 1 Department of Orthopedic Surgery, Chung-Ang University Gwangmyeong Hospital, Chung-Ang University College of Medicine, Seoul 14353, Republic of Korea; whybe1122@gmail.com; 2 Department of Orthopedic Surgery, Hallym Sacred Heart University Hospital, Hallym University, Anyang-si 13496, Republic of Korea

**Text Correction**

There was an error in the original publication [1]. We would like to request a minor correction in the article above. On page 7, the values were misprinted, although the values in the figure (forest plot) were correct.

A correction has been made to the Result section, 3.3.2. Functional Improvement.

3.3.2. Functional Improvement

In the short-term follow-up, ten celecoxib [18,31,33–36,39,43,44,46] and two JOINS [12,18] cohorts reported changes in the total WOMAC score from the preoperative to postoperative period. The meta-analysis-estimated functional improvement of the total WOMAC score was 23.1 (95% CI, 18.8–27.4) in the celecoxib and 13.9 (95% CI, 7.1–20.6) in the JOINS cohorts, which indicated no significant difference between the two cohorts (*p* = 0.159) (Figure 3A). In the mid-term follow-up, seven celecoxib [18,30,32,37,40,41,44] and three JOINS [12,17,18] cohorts reported a mean improvement in total WOMAC score of 20.3 (95% CI, 13.8–26.9) and 17.0 (95% CI, 12.2–21.7), respectively, which indicated no significant difference between the two cohorts (*p* = 0.451) (Figure 3B).

The authors state that the scientific conclusions are unaffected. This correction was approved by the Academic Editor. The original publication has also been updated.
