# Supplementary material for: Efficacy and Safety of Celecoxib and a Korean SYSADOA (JOINS) for the Treatment of Knee Osteoarthritis: A Systematic Review and Meta-Analysis
Source: J Clin Med. 2025 Feb 7;14(4):1036. doi: 10.3390/jcm14041036 (PMC11856201; doi:10.3390/jcm14041036)
Supplement: Supplementary file 1 [file jcm-14-01036-s001.zip › jcm-3409133-supplementary.pdf]

Supplementary Table S1. A summary of the MINORS score in the included studies in the meta-analysis.

Study (year)	1	2	3	4	5	6	7	8	9	10	11	12	MINORS score†
**Celecoxib Group**													
Bin et al. (2024) [18]	2	2	2	2	2	2	2	2	2	2	2	2	24
Clegg et al. (2006) [30]	2	2	2	2	2	2	1	2	2	2	2	2	23
Essex et al. (2016) [34]	2	2	2	2	2	2	2	2	2	2	2	2	24
Essex et al. (2014) [31]	2	2	2	2	2	2	2	2	2	2	2	2	24
Essex et al. (2012) [32]	2	2	2	2	1	2	1	2	2	2	2	2	22
Essex et al. (2012) [33]	2	2	2	2	2	2	1	2	2	2	2	2	23
Geba et al. (2002) [22]	2	2	2	2	2	2	2	2	2	2	2	2	24
Gibofsky et al. (2003) [35]	2	2	2	2	2	2	2	2	2	2	2	2	24
Gordo et al. (2017) [36]	2	2	2	2	2	2	2	2	2	2	2	2	24
Hochberg et al. (2016) [37]	2	2	2	2	2	2	1	2	2	2	2	2	23
Jin et al. (2018) [38]	2	2	2	2	2	2	1	2	2	2	2	2	23
Park et al. (2013) [39]	2	2	2	2	2	2	1	2	2	2	2	2	23
Pelletier et al. (2020) [41]	2	2	2	2	2	2	1	2	2	2	2	2	23
Pelletier et al. (2016) [40]	2	2	2	2	2	2	1	2	2	2	2	2	23
Reginster et al. (2017) [42]	2	2	2	1	2	2	1	2	2	2	2	2	22
Williams et al. (2001) [43]	2	2	2	1	2	2	1	2	2	2	2	1	21
Xu et al. (2023) [44]	2	2	2	2	2	2	1	2	2	2	2	2	23
Yoo MC et al. (2014) [45]	2	2	2	2	2	2	2	2	2	2	2	2	23
Yoo WH et al. (2014) [46]	2	2	2	2	2	2	2	2	2	2	2	2	23
**JOINS Group**													
Bin et al. (2024) [18]	2	2	2	2	2	2	1	2	2	2	2	2	23
Ha et al. (2016) [12]	2	2	2	2	2	2	2	2	2	2	2	2	24
Jung et al. (2001) [15]	2	2	2	2	2	2	1	2	2	2	2	2	23
Jung et al. (2004) [19]	2	2	2	2	1	2	1	2	2	2	2	2	22
Kim et al. (2017) [17]	2	2	2	2	2	2	1	2	2	2	2	2	23

†MINORS, Methodological Index for Non-Randomized Studies

1. A clearly stated aim, 2. Inclusion of consecutive patients, 3. Prospective collection of data, 4. Endpoints appropriate to the aim of the study, 5. Unbiased assessment of the study endpoint, 6. Follow-up period appropriate to the aim of the study, 7. Loss to follow up less than 5%, 8. Prospective calculation of the study size, 9. An adequate control group, 10. Contemporary groups, 11. Baseline equivalence of groups, 12. Adequate statistical analyses.

Supplementary Table S2. A summary of the bias of RCTs included in the meta-analysis.

Study (year)	Random sequence generation (selection bias)	Allocation concealment (selection bias)	Blinding of participants and personnel (performance bias)	Blinding of outcome assessment (detection bias)	Incomplete outcome data (attrition bias)	Selective reporting (reporting bias)	Other bias
Bin et al. (2024) [18]	low	unclear	low	low	low	low	low
Clegg et al. (2006) [30]	low	low	low	unclear	low	low	low
Essex et al. (2016) [34]	low	unclear	low	unclear	low	low	low
Essex et al. (2014) [31]	unclear	unclear	low	unclear	low	low	low
Essex et al. (2012) [32]	unclear	unclear	low	unclear	low	low	low
Essex et al. (2012) [33]	unclear	unclear	low	unclear	low	low	low
Geba et al. (2002) [22]	unclear	unclear	low	unclear	low	low	unclear
Gibofsky et al. (2003) [35]	unclear	unclear	low	unclear	low	unclear	high
Gordo et al. (2017) [36]	low	low	low	unclear	low	low	low
Ha et al. (2016) [12]	low	low	low	unclear	low	low	low
Hochberg et al. (2016) [37]	low	low	low	low	low	low	low
Jin et al. (2018) [38]	low	low	low	unclear	low	low	low
Jung et al. (2001) [15]	low	unclear	low	high	unclear	low	unclear
Jung et al. (2004) [19]	low	low	low	low	low	low	low
Kim et al. (2017) [17]	low	low	low	unclear	unclear	low	low
Park et al. (2013) [39]	low	low	low	unclear	low	low	low
Pelletier et al. (2020) [41]	low	low	low	unclear	low	low	low

Pelletier et al. (2016) [40]	low	low	low	low	low	low	unclear
Reginster et al. (2017) [42]	unclear	low	unclear	unclear	low	unclear	unclear
Williams et al. (2001) [43]	unclear	unclear	low	unclear	low	low	unclear
Xu et al. (2023) [44]	low	low	low	unclear	unclear	low	low
Yoo MC et al. (2014) [45]	low	low	low	unclear	unclear	low	low
Yoo WH et al. (2014) [46]	low	low	low	unclear	unclear	low	low

References

12. Ha, C.W.; Park, Y.B.; Min, B.W.; Han, S.B.; Lee, J.H.; Won, Y.Y.; Park, Y.S. Prospective, randomized, double-blinded, double-dummy and multicenter phase IV clinical study comparing the efficacy and safety of PG201 (Layla) and SKI306X in patients with osteoarthritis. *J. Ethnopharmacol.* **2016**, *181*, 1–7. https://doi.org/10.1016/j.jep.2016.01.029.
15. Jung, Y.B.; Roh, K.J.; Jung, J.A.; Jung, K.; Yoo, H.; Cho, Y.B.; Kwak, W.J.; Kim, D.K.; Kim, K.H.; Han, C.K. Effect of SKI 306X, a new herbal anti-arthritic agent, in patients with osteoarthritis of the knee: A double-blind placebo controlled study. *Am. J. Chin. Med.* **2001**, *29*, 485–491. https://doi.org/10.1142/S0192415X01000502.
17. Kim, J.I.; Choi, J.Y.; Kim, K.G.; Lee, M.C. Efficacy of JOINS on Cartilage Protection in Knee Osteoarthritis: Prospective Randomized Controlled Trial. *Knee Surg. Relat. Res.* **2017**, *29*, 217–224. https://doi.org/10.5792/ksrr.17.004.
18. Bin, S.I.; Lee, M.C.; Kang, S.B.; Moon, Y.W.; Yoon, K.H.; Han, S.B.; In, Y.; Chang, C.B.; Bae, K.C.; Sim, J.A.; et al. Efficacy and safety of SKCPT in patients with knee osteoarthritis: A multicenter, randomized, double-blinded, active-controlled phase III clinical trial. *J. Ethnopharmacol.* **2024**, *337*, 118843. https://doi.org/10.1016/j.jep.2024.118843.
19. Jung, Y.B.; Seong, S.C.; Lee, M.C.; Shin, Y.U.; Kim, D.H.; Kim, J.M.; Jung, Y.K.; Ahn, J.H.; Seo, J.G.; Park, Y.S.; et al. A four-week, randomized, double-blind trial of the efficacy and safety of SKI306X: A herbal anti-arthritic agent versus diclofenac in osteoarthritis of the knee. *Am. J. Chin. Med.* **2004**, *32*, 291–301. https://doi.org/10.1142/S0192415X04001941.
22. Geba, G.P.; Weaver, A.L.; Polis, A.B.; Dixon, M.E.; Schnitzer, T.J. Efficacy of rofecoxib, celecoxib, and acetaminophen in osteoarthritis of the knee: A randomized trial. *JAMA* **2002**, *287*, 64–71. https://doi.org/10.1001/jama.287.1.64.
30. Clegg, D.O.; Reda, D.J.; Harris, C.L.; Klein, M.A.; O'Dell, J.R.; Hooper, M.M.; Bradley, J.D.; Bingham, C.O., 3rd; Weisman, M.H.; Jackson, C.G.; et al. Glucosamine, chondroitin sulfate, and the two in combination for painful knee osteoarthritis. *N. Engl. J. Med.* **2006**, *354*, 795–808. https://doi.org/10.1056/NEJMoa052771.
31. Essex, M.N.; Behar, R.; O'Connell, M.A.; Brown, P.B. Efficacy and tolerability of celecoxib and naproxen versus placebo in Hispanic patients with knee osteoarthritis. *Int. J. Gen. Med.* **2014**, *7*, 227–235. https://doi.org/10.2147/IJGM.S61297.
32. Essex, M.N.; Bhadra, P.; Sands, G.H. Efficacy and tolerability of celecoxib versus naproxen in patients with osteoarthritis of the knee: A randomized, double-blind, Double-dummy Trial. *J. Int. Med. Res.* **2012**, *40*, 1357–1370. https://doi.org/10.1177/147323001204000414.

33. Essex, M.N.; O'Connell, M.; Bhadra Brown, P. Response to nonsteroidal anti-inflammatory drugs in African Americans with osteoarthritis of the knee. *J. Int. Med. Res.* **2012**, *40*, 2251–2266. https://doi.org/10.1177/030006051204000623.
34. Essex, M.N.; O'Connell, M.A.; Behar, R.; Bao, W. Efficacy and safety of nonsteroidal anti-inflammatory drugs in Asian patients with knee osteoarthritis: Summary of a randomized, placebo-controlled study. *Int. J. Rheum Dis.* **2016**, *19*, 262–270. https://doi.org/10.1111/1756-185x.12667.
35. Gibofsky, A.; Williams, G.W.; McKenna, F.; Fort, J.G. Comparing the efficacy of cyclooxygenase 2-specific inhibitors in treating osteoarthritis: Appropriate trial design considerations and results of a randomized, placebo-controlled trial. *Arthritis Rheum* **2003**, *48*, 3102–3111. https://doi.org/10.1002/art.11330.
36. Gordo, A.C.; Walker, C.; Armada, B.; Zhou, D. Efficacy of celecoxib versus ibuprofen for the treatment of patients with osteoarthritis of the knee: A randomized double-blind, non-inferiority trial. *J. Int. Med. Res.* **2017**, *45*, 59–74. https://doi.org/10.1177/0300060516673707.
37. Hochberg, M.C.; Martel-Pelletier, J.; Monfort, J.; Möller, I.; Castillo, J.R.; Arden, N.; Berenbaum, F.; Blanco, F.J.; Conaghan, P.G.; Doménech, G.; et al. Combined chondroitin sulfate and glucosamine for painful knee osteoarthritis: A multicentre, randomised, double-blind, non-inferiority trial versus celecoxib. *Ann. Rheum Dis.* **2016**, *75*, 37–44. https://doi.org/10.1136/annrheumdis-2014-206792.
38. Jin, Y.; Smith, C.; Monteith, D.; Brown, R.; Camporeale, A.; McNearney, T.A.; Deeg, M.A.; Raddad, E.; Xiao, N.; de la Peña, A.; et al. CGRP blockade by galcanezumab was not associated with reductions in signs and symptoms of knee osteoarthritis in a randomized clinical trial. *Osteoarthr. Cartil.* **2018**, *26*, 1609–1618. https://doi.org/10.1016/j.joca.2018.08.019.
39. Park, Y.G.; Ha, C.W.; Han, C.D.; Bin, S.I.; Kim, H.C.; Jung, Y.B.; Lim, H.C. A prospective, randomized, double-blind, multicenter comparative study on the safety and efficacy of Celecoxib and GCSB-5, dried extracts of six herbs, for the treatment of osteoarthritis of knee joint. *J. Ethnopharmacol.* **2013**, *149*, 816–824. https://doi.org/10.1016/j.jep.2013.08.008.
40. Pelletier, J.P.; Raynauld, J.P.; Beaulieu, A.D.; Bessette, L.; Morin, F.; de Brum-Fernandes, A.J.; Delorme, P.; Dorais, M.; Paiement, P.; Abram, F.; et al. Chondroitin sulfate efficacy versus celecoxib on knee osteoarthritis structural changes using magnetic resonance imaging: A 2-year multicentre exploratory study. *Arthritis Res. Ther.* **2016**, *18*, 256. https://doi.org/10.1186/s13075-016-1149-0.
41. Pelletier, J.P.; Raynauld, J.P.; Dorais, M.; Bessette, L.; Dokoupilova, E.; Morin, F.; Pavelka, K.; Paiement, P.; Martel-Pelletier, J. An international, multicentre, double-blind, randomized study (DISSCO): Effect of diacerein vs celecoxib on symptoms in knee osteoarthritis. *Rheumatology* **2020**, *59*, 3858–3868. https://doi.org/10.1093/rheumatology/keaa072.
42. Reginster, J.Y.; Dudler, J.; Blicharski, T.; Pavelka, K. Pharmaceutical-grade Chondroitin sulfate is as effective as celecoxib and superior to placebo in symptomatic knee osteoarthritis: The ChONdroitin versus CElecoxib versus Placebo Trial (CONCEPT). *Ann. Rheum Dis.* **2017**, *76*, 1537–1543. https://doi.org/10.1136/annrheumdis-2016-210860.
43. Williams, G.W.; Ettlinger, R.E.; Ruderman, E.M.; Hubbard, R.C.; Lonien, M.E.; Yu, S.S.; Zhao, W.; Geis, G.S. Treatment of osteoarthritis with a once-daily dosing regimen of celecoxib: A randomized, controlled trial. *J. Clin. Rheumatol.* **2000**, *6*, 65–74. https://doi.org/10.1097/00124743-200004000-00002.
44. Xu, H.; Zhao, C.; Guo, G.; Li, Y.; A, X.; Qiu, G.; Wang, Y.; Kang, B.; Xu, X.; Xie, J.; et al. The Effectiveness of Tuina in Relieving Pain, Negative Emotions, and Disability in Knee Osteoarthritis: A Randomized Controlled Trial. *Pain Med.* **2023**, *24*, 244–257. https://doi.org/10.1093/pm/pnac127.
45. Yoo, M.C.; Yoo, W.H.; Kang, S.B.; Park, Y.W.; Kim, S.S.; Moon, K.H.; Song, Y.W.; Min, B.W.; Cho, Y.J.; Moon, S.H.; et al. Etoricoxib in the treatment of Korean patients with osteoarthritis in a double-blind, randomized controlled trial. *Curr. Med. Res. Opin.* **2014**, *30*, 2399–2408. https://doi.org/10.1185/03007995.2014.955169.
46. Yoo, W.H.; Yoo, H.G.; Park, S.H.; Baek, H.J.; Lee, Y.J.; Shim, S.C.; Kang, S.W.; Kim, H.A.; Song, J.S.; Suh, C.H.; et al. Efficacy and safety of PG201 (Layla(®)) and celecoxib in the treatment of symptomatic knee osteoarthritis: A double-blinded, randomized, multi-center, active drug comparative, parallel-group, non-inferiority, phase III study. *Rheumatol. Int.* **2014**, *34*, 1369–1378. https://doi.org/10.1007/s00296-014-2964-8.
